# Quantification of Live Bacterial Sensing for Chemotaxis and Phagocytosis and of Macropinocytosis

**DOI:** 10.3389/fcimb.2018.00062

**Published:** 2018-03-02

**Authors:** Netra P. Meena, Alan R. Kimmel

**Affiliations:** Laboratory of Cellular and Developmental Biology, National Institute of Diabetes and Digestive and Kidney Diseases, The National Institutes of Health, Bethesda, MD, United States

**Keywords:** cell migration, *Bacillus*, *Pseudomonas*, *E*. *coli*, pterin, cAMP, innate immunity, *Dictyostelium*

## Abstract

Initial immunological defense mechanisms to pathogen invasion rely on innate pathways of chemotaxis and phagocytosis, original to ancient phagocytes. Although chemotaxis has been well-studied in mammalian and model systems using purified chemoattractants in defined conditions, directed movement toward live bacteria has been more difficult to assess. *Dictyostelium discoideum* is a professional phagocyte that chemotaxes toward bacteria during growth-phase in a process to locate nutrient sources. Using *Dictyostelium* as a model, we have developed a system that is able to quantify chemotaxis to very high sensitivity. Here, *Dictyostelium* can detect various chemoattractants at concentrations <1 nM. Given this exceedingly sensitive signal response, *Dictyostelium* will migrate directionally toward live gram positive and gram negative bacteria, in a highly quantifiable manner, and dependent upon bacterially-secreted chemoattractants. Additionally, we have developed a real-time, quantitative assay for phagocytosis of live gram positive and gram negative bacteria. To extend the analyses of endocytic functions, we further modified the system to quantify cellular uptake *via* macropinocytosis of smaller (<100 kDa) molecules. These various approaches provide novel means to dissect potential for identification of novel chemoattractants and mechanistic factors that are essential for chemotaxis, phagocytosis, and/or macropinocytosis and for more detailed understanding in host-pathogen interactive defenses.

## 1. Introduction

Host–pathogen interactions are crucial aspects to the immune response. Innate immunity comprises generalized defense to invading microorganisms and involves cellular functions, including chemotaxis and phagocytosis, that can be traced at least 1 billion years (Cosson and Soldati, 2008; Jin et al., 2008; Bozzaro and Eichinger, 2011), where ancient professional phagocytes utilized the pathways in a search for and an uptake of new nutrient sources. Thus, similar molecular processes, including receptor signaling and directed cytoskeletal mobilization, are essential for both destruction of pathogenic bacteria by macrophage and neutrophils, and for nutrient capture by *Dictyostelium discoideum* (Cosson and Soldati, 2008; Jin et al., 2008; Bozzaro and Eichinger, 2011). Significantly, the ease in genetic manipulation of *Dictyostelium* (Basu et al., 2013) has made it an ideal model for mechanistic studies of these processes.

*Dictyostelium* can rapidly sense multiple chemoattractants in very broad and dynamic concentration gradients (Jin, 2013; Artemenko et al., 2014; Nichols et al., 2015). We established a new quantitative system for high-throughput chemoattractant gradient perception at very low concentration (<1 nM) sensitivity and then extended conditions for use of live bacteria as the source for secreted chemoattractants (Meena and Kimmel, 2017). This further permitted analyses to understand simultaneous response to multiple chemoattractants and to identify bacterially secreted factors. Beyond, we have modified the system to examine functions secondary to chemotaxis that are essential for target removal through phagocytosis and macropinocytosis.

Procedural details are, thus, presented for quantification of chemotaxis in real-time to both purified ligands, individually, or in multiple combinations, or to live bacteria. We also outline specific approaches for quantifying cellular uptake of large (>1 μm) particles by phagocytosis or of molecules (<100 kDa) by macropinocytosis, again in a real-time and high-throughput mode.

## 2. Materials and equipment

### 2.1. Solutions

1. *Dictyostelium* growth medium: D3-T Medium KD Medical, cat # D3-T001 (Composition/1L: 7.15 g proteose peptone, 7.15 g peptone A, 7.15 g yeast extract, 15.3 g glucose, 0.52 g Na_2_HPO_4_^*^7H_2_O, 0.48 g KH_2_PO_4_ – pH 6.4).
2. Phosphate Buffer (PB): 5 mM Na_2_HPO_4_, 5 mM NaH_2_PO_4_, adjusted to pH 6.5.
3. cAMP: 1 mM in PB, dilute as needed.
4. Folate, Pterin, and Methopterin: 50 mM in PB, neutralized as needed with 1 N NaOH. All of these compounds are light sensitive; for most consistent results, prepare fresh and keep in a light-tight vial.
5. LB medium: KD Medical, cat # BLF-7030.
6. Nutrient broth: BD, cat # 234000.
7. Tryptic soya broth: BD, cat # 211825.

### 2.2. Supplies

1. *Dictyostelium discoideum*, strain Ax3: dictyBase (http://dictybase.org).
2. Shaker Incubator: New Brunswick Innova 44 incubator shaker.
3. The IncuCyte® Live Cell Analysis System: Essen Bioscience, cat # 4582.
4. *Escherichia coli*, Strain *Dh5*α: Thermo Fisher Scientific, cat # C404010.
5. *Bacillus cereus*: ATCC® 14579™.
6. *Micrococcus luteus*: ATCC® 4698™.
7. *Pseudomonas fluorescens*: ATCC® 31086™.
8. pHrodo™ Red Dextran, 10,000 MW: ThermoFisher Scientific, cat # P10361.
9. Spectrophotometer: GE Healthcare/Amersham Biosciences Ultrospec 3100 Pro UV/Visible Spectrophotometer # 80-2112-31.
10. Lab-TeK 4-well-chambered slide: ThermoFisher Scientific, cat # 155383.
11. Lab-TeK 8-well-chambered slide: ThermoFisher Scientific, cat # 155411.
12. Confocal Microscope: LSM 780 confocal/multiphoton microscope, Carl Zeiss, Thornwood, NY, USA.
13. Chemotaxis 96 well plate: Essen BioScience, cat # 4648.
14. 96 well, flat bottom plate for phagocytosis and macropinocytosis: Corning, cat # 3596.
15. Bovine serum albumin (BSA): Sigma-Aldrich, cat # A3803.
16. pHrodo^TM^ Phagocytosis particle labeling kit: Thermo Fisher Scientific, cat # A10026*7*.

## 3. Procedures and results

### 3.1. Chemotaxis by growth phase *Dictyostelium* to chemoattractants, folate, methopterin, pterin, and cAMP

#### 3.1.1. Growth and preparation of dictyostelium

**3.1.1.1.** Grow *Dictyostelium* in D3-T Medium at 22°C in shaking culture, at ~200 rpm, to 1 × 10^6^ cells/mL.
**3.1.1.2.** Centrifuge cells at 22°C for 5 min at 500 × *g*.
**3.1.1.3.** Wash cells once with 10 mM phosphate buffer (PB), pH 6.5 at 22°C.
**3.1.1.4.** Resuspend cells at 1.5–4.5 × 10^4^ cells/mL in PB, by gentle tapping of the bottom of tube.

#### 3.1.2. Quantification of dictyostelium chemotaxis to chemical ligands

**3.1.2.1.** Coat both the upper and lower chambers of the chemotaxis plates with 1% Bovine serum albumin (BSA) in PB, adding 60 μL of 1% BSA to top chamber and 160 μL to the bottom well. Incubate the plates at room temperature.
**3.1.2.2.** After 1 h, temporarily move the upper part of the plate to a new plate bottom and carefully remove the BSA from each well of the upper plate chamber without touching the membrane.
**3.1.2.3.** Add 60 μL (1–3 × 10^3^) of growing *Dictyostelium* (from **3.1.1.4**.) in PB to the upper well of a BSA-treated chemotaxis plate. Avoid creating air bubbles at the surface of the membrane.
**3.1.2.4.** Completely remove BSA from each well of the BSA-treated bottom plate and add 200 μL PB with or without chemoattractants at desired concentrations (e.g., 0–1 mM).
**3.1.2.5.** After adding both cells and chemoattractants to their respective parts of the chemotaxis plate, reassemble the original plate. Carefully place the upper part of the chemotaxis plate on top of the lower part of the plate, and avoid generating any air bubbles in the process.
**3.1.2.6.** Transfer the assembled plate into the IncuCyte chamber, at 22°C. Using a 10x objective lens, collect cell images from both the top and under surface of the membrane, over a desired time (e.g., 1.5–4 h), at 15 min intervals. Export the data into Microsoft Excel for processing.

#### 3.1.3. Results

*Dictyostelium* grow as single cells that migrate to bacterially secreted pterin-derived folate moieties and to cAMP (Meena and Kimmel, 2017), in a search for nutrient sources. Assaying chemotaxis of growth-phase *Dictyostelium* had been a great challenge (Veltman et al., 2014). However, we developed a highly sensitive, quantitative, and reproducible method for chemotaxis of growth-phase *Dictyostelium* that is easily modified and optimized for most single-cell types (Meena and Kimmel, 2017).

The approach adapts the IncuCyte® live-cell analysis system from Essen Bioscience for real-time imaging of chemotaxis in a 96-well format. IncuCyte® uses high definition phase contrast imaging and ClearView Cell Migration Plates. The scan intervals, digital zoom, and imaging channels are set manually. But following, image acquisition is fully automated, as are integrated real-time data analyses and full time-course metrics, with application of the IncuCyte® Chemotaxis Cell Migration Software Module. The specialized 96-well chemotaxis dish is comprised of three sections: a transparent lid, an upper well plate (which will contain cells to be assayed), and a bottom well plate (which will have chemoattractants). A membrane of uniformly-spaced 8 μm pores separates each aligned upper and bottom chamber well.

**Figure 1:**
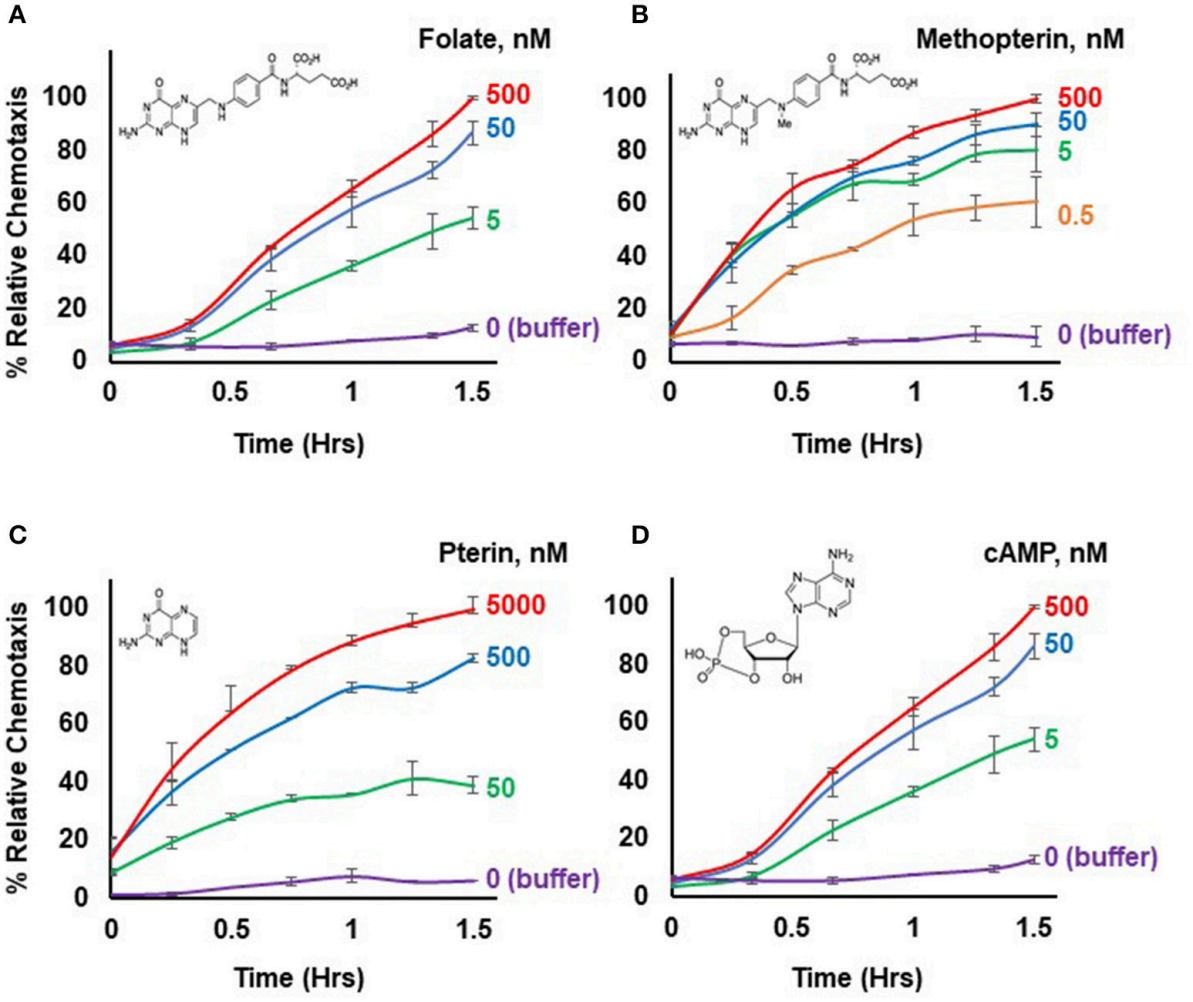
Chemotactic dose-responses to different chemoattractants. **(A)** Time-course quantification of *Dictyostelium* migration to various dose concentrations of folate. Relative chemotaxis is normalized to 500 nM folate at 1.5 h; no additional chemotaxis is observed with folate increased to 5,000 nM (see Figure 2). Standard deviations are shown based upon three replicates. The structure of folate is shown. **(B)** Time-course quantification of *Dictyostelium* migration to various dose concentrations of methopterin. Relative chemotaxis is normalized to 500 nM methopterin at 1.5 h. Standard deviations are shown based upon three replicates. The structure of methopterin is shown. **(C)** Time-course quantification of *Dictyostelium* migration to various dose concentrations of pterin. Relative chemotaxis is normalized to 5,000 nM pterin at 1.5 h. Standard deviations are shown based upon three replicates. The structure of pterin is shown. **(D)** Time-course quantification of *Dictyostelium* migration to various dose concentrations of cAMP. Relative chemotaxis is normalized to 500 nM cAMP at 1.5 h; no additional chemotaxis is observed with cAMP increased to 5,000 nM (see Figure 2). Standard deviations are shown based upon three replicates. The structure of cAMP is shown.

Chemoattractant gradients are established by chemical diffusion from the bottom wells, through the membrane pores, into the upper wells. Cell migration results from the movement of cells out of the upper well, through the membrane toward the bottom well, and chemotaxis is quantified over time, by imaging cells that had migrated from the upper well base to the under surface of the membrane within the bottom chamber. The assays are highly reproducible, with generally <10% non-specific directed migration to phosphate buffer controls [Figure 1 (Meena and Kimmel, 2017)]. In addition, by comparing migration toward buffer controls, using cells incubated in the presence or absence of chemoattractants, we can assess a potential chemokinetic contribution to random migration (Meena and Kimmel, 2017). Chemokinesis may increase operative backgrounds to ~15%, for fully responsive cells (Meena and Kimmel, 2017).

**Figure 2:**
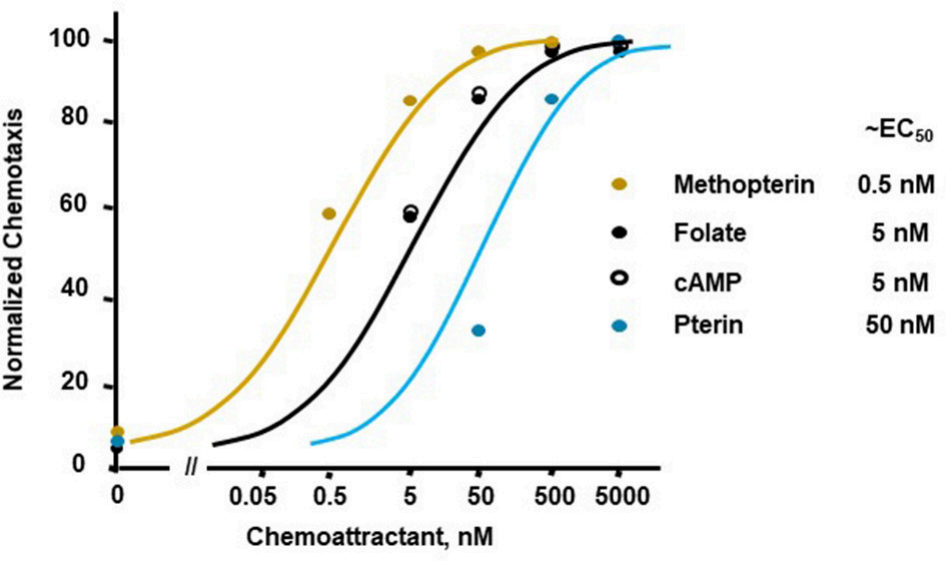
Quantified dose-responses for *Dictyostelium* chemotaxis to different chemoattractants. Curve fits are shown for an indicated EC_50_. Symbols presented are means from three separate experiments at 1.5 h, as derived from data as per Figure 1.

Figure 1 illustrates a typical chemotactic dose-response, time-course for migration of growth-phase *Dictyostelium* toward four different chemoattractants. Folate, methopterin, and pterin represent 3 structurally related moieties that interact with the same chemoattractant receptor (NPM and ARK, in preparation), but at differing activating affinities, in the order of methopterin>folate>pterin (NPM and ARK, in preparation); the EC_50_ for chemotaxis of each is consistent with these affinity differences (Figure 2). Separately, we also show high chemosensitivity of growth-phase *Dictyostelium* to the unrelated chemoattractant cAMP (Figures 1D, 2), which interacts with a distinct chemoattractant receptor (Liao et al., 2013; Meena and Kimmel, 2016, 2017).

### 3.2. Chemotaxis of growth phase *Dictyostelium* to live bacteria

#### 3.2.1. Growth and preparation of growth phase dictyostelium

Follow all steps in **3.1.1**.

#### 3.2.2. Growth of bacteria

**3.2.2.1.** Grow *Escherichia coli* in LB medium at 37°C, in shaking culture at ~200 rpm, to OD_600_ <1.
**3.2.2.2.** Grow *Bacillus cereus* in Nutrient broth medium at 30°C, in shaking culture at ~200 rpm, to OD_600_ <1.
**3.2.2.3.** Grow *Pseudomonas fluorescens* in Nutrient broth medium at 28°C, in shaking culture at ~200 rpm, to OD_600_ <1.
**3.2.2.4.** Grow *Micrococcus luteus* in Tryptic soya broth medium at 30°C, in shaking culture at ~200 rpm, to OD_600_ <1.

#### 3.2.3. Preparation of bacteria for chemotaxis

**3.2.3.1.** Wash bacteria three times in PB, with centrifugation at ~6,000 rpm, room temperature for 2 min.
**3.2.3.2.** Resuspend bacteria in PB at 2.5 × 10^8^ cells/ml.

#### 3.2.4. Quantification of chemotaxis using bacteria as signaling agents

**3.2.4.1.** Prepare chemotaxis plates with *Dictyostelium* in the upper chamber wells following steps **3.1.2.1–3.1.2.3**.
**3.2.4.2.** Remove BSA completely from each well of the treated bottom plate and add PB with bacteria at the desired cell number (e.g., 0–50 × 10^6^ bacteria) to the bottom well of the plate.
**3.2.4.3.** After adding both *Dictyostelium* and bacteria to their respective parts of the chemotaxis plate, reassemble the original plate. Carefully place upper part of the plate on top of the lower part of the chemotaxis plate, avoid generating any air bubble in this process.
**3.2.4.4.** Transfer the assembled plate into the IncuCyte chamber, at 22°C. Using a 10x objective lens, collect cell images from both the top, and under surface of the membrane, over a desired time (e.g., 1.5–4 h.), at 15 min intervals. Export the data into Microsoft Excel for processing.

#### 3.2.5. Results

The response sensitivity of growth-phase *Dictyostelium* to <1 nM concentrations of various chemoattractants suggested that the assay might be sufficiently effective to detect directed migration to live bacteria secreting endogenous compounds, which act as chemoattractants to *Dictyostelium*. Indeed, we had previously demonstrated that *Dictyostelium* would chemotax to both *Klebsiela planticola* and *Bacillus subtilis* (Meena and Kimmel, 2017). Relative chemotaxis was directly correlated to cell number, indicating an excellent dose-dependent quantifiable response. We also showed that chemotaxis relied on folate- and/or cAMP-dependent signaling pathways and that secreted cAMP could be detected by bioassay (Meena and Kimmel, 2017).

**Figure 3:**
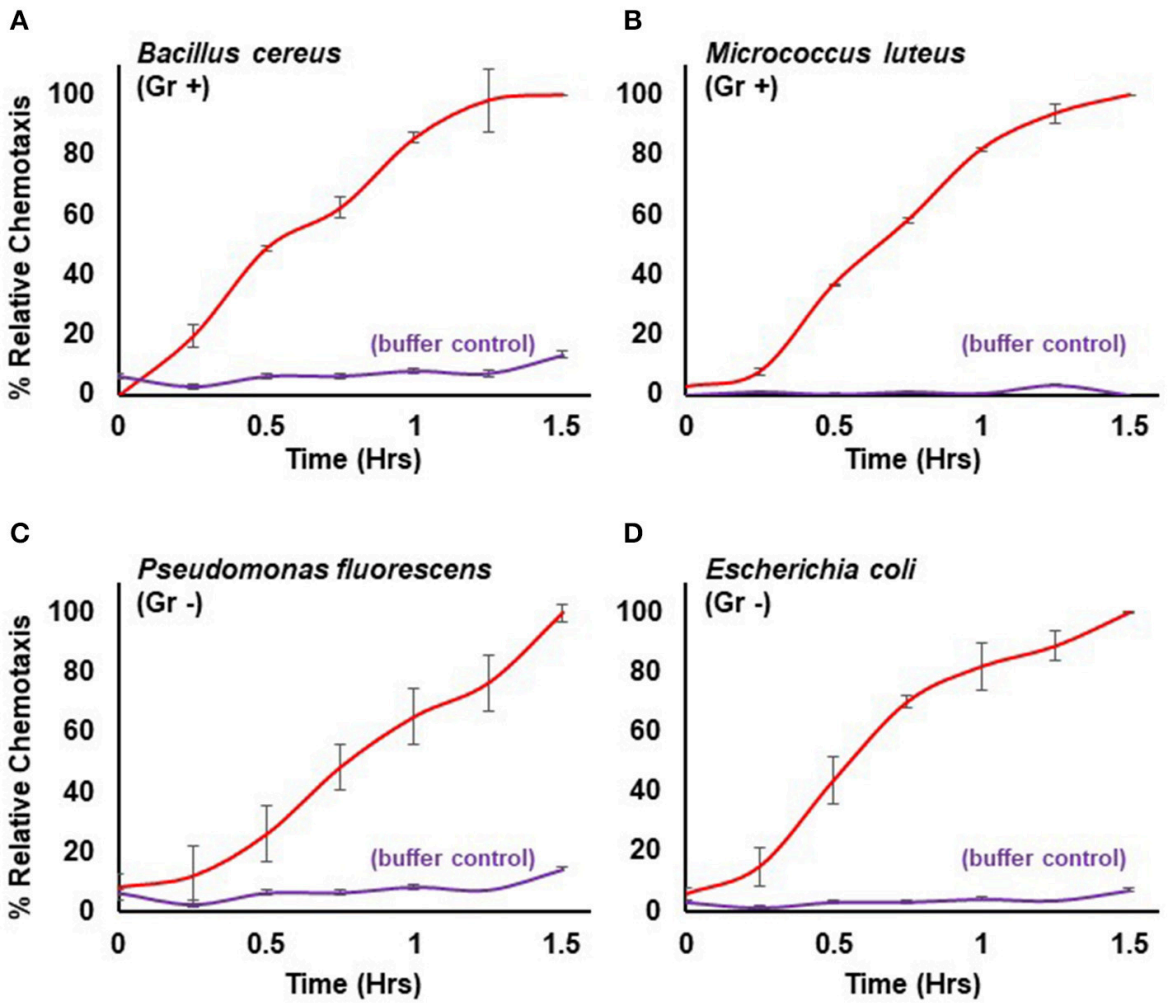
Quantified chemotaxis to live bacteria. Time-course quantification of WT *Dictyostelium* migration to 5 × 10^7^ cells of **(A)** gram positive (Gr +) *Bacillus cereus*, **(B)** gram positive (Gr+) *Micrococcus luteus*, **(C)** gram negative (Gr -) *Pseudomonas fluorescens*, and **(D)** gram negative (Gr–) *Escherichia coli*; buffer controls are included for each. Relative chemotaxis is normalized to values at 1.5 h. Standard deviations are shown based upon three replicates.

Here, we extend the system to define conditions for quantifiable chemotaxis to other live bacterial species. *Dictyostelium* show strong chemotactic response to both gram positive (*Bacillus cereus, Micrococcus luteus*) and gram negative (*Escherichia coli, Pseudomonas fluorescens*) bacteria (Figure 3), in a manner similar to that of purified chemoattractants (see Figure 1). Physio-chemical analyses confirm the secretion of cAMP and/or folate, by the various bacteria.

### 3.3. Phagocytosis of live bacteria by growth phase *Dictyostelium*

#### 3.3.1. Growth and preparation of growth phase dictyostelium

Follow all steps in **3.1.1**.

#### 3.3.2. Growth and preparation of bacteria

Follow all steps in **3.2.2**. and **3.2.3**.

#### 3.3.3. Preparation of pHrodo®-labeled live bacteria

**3.3.3.1.** pHrodo^TM^ is a fluorogenic dye that exhibits a dramatic increase in fluorescence at an acidified pH. Thus, pHrodo®-labeled bacteria show minimal basal fluorescence, but upon phagocytosis by *Dictyostelium* and transport to lysosomes, detected fluorescence increases. ~2 x 10^9^ bacteria from **3.2.3**. were centrifuged for 2 min at 12,000 rpm and resuspended in ~50 μL of Component C (ThermoFisher Scientific, pHrodo^TM^ Phagocytosis particle labeling kit). Succinimidyl ester dye (10 mM) was added to 0.5 mM and incubated for 45 min at room temperature. To prevent cell lethality during pHrodo®-labeling, toxic compounds (e.g., methanol) were eliminated from the labeling.
**3.3.3.2.** To remove non-complexed dye, bacteria were washed three times at 12,000 rpm with Component C. All pHrodo®-labeled bacteria species were viable, as observed by cell division and motility, when re-cultured in growth medium.

#### 3.3.4. Quantification of phagocytosis of live bacteria

**3.3.4.1.** ~0.9 x 10^5^ *Dictyostelium* from **3.1.2.4**. in 100 μL PB were added to each phagocytosis/macropinocytosis plate well and allowed to adhere to the well base for 1–5 min.
**3.3.4.2.** ~1 x 10^7^ (~10-fold the number of *Dictyostelium* cells) of pHrodo®-labeled live bacteria from **3.3.3.2**. in 100 μL of PB were added to each well. In parallel, for negative controls, three wells had bacteria, without added *Dictyostelium*.
**3.3.4.3.** Transfer the plate to the IncuCyte chamber, at 22°C. Using a 10x objective lens, collect cell images in the visible and rhodamine detection ([Excitation/Emission (nm) 560/585]), a desired time (e.g., 1.5–4 h), at 15 min intervals. Export the data into Microsoft Excel for processing.

#### 3.3.5. Phagocytosis of pHrodo-labeled bacteria using immunofluorescence microscopy

**3.3.5.1.** Follow all steps in **3.1.1**. to obtain growth phase *Dictyostelium*.
**3.3.5.2.** Follow all steps in **3.3.3**. to obtain pHrodo-labeled bacteria.
**3.3.5.3.** Phagocytosis of pHrodo-labeled bacteria by *Dictyostelium* can also be visualized by confocal microscopy. Briefly, add 400 μL of pHrodo-labeled live bacteria 8 × 10^6^ /mL in PB and 400 μL of *Dictyostelium* at 1.0 x 10^6^ /mL in PB to a well of a 4-well Lab-Tec chambered slide and incubate at 22°C for 15 min, to allow cells to adhere.
**3.3.5.4.** DIC and fluorescent [Excitation/Emission (nm) 560/585] images were acquired at varying times by confocal/multiphoton microscopy using a 100x oil objective lens and processed using Zeiss ZEN software.

#### 3.3.6. Results

In addition to chemotaxis, professional phagocytes are defined by an ability to engulf large (>1 μM) particles, for nutrient uptake, directed killing of invading unicellular organisms, and/or removal of cell debris (Lim et al., 2017). Essential to phagocytic processes is the acidification of phagosomes through a vesicle trafficking pathway leading to foreign particle destruction (Levin et al., 2016). We have taken advantage of the rhodamine probe pHrodo® that has minimal fluorescence in a pH neutral milieu, but which displays enhanced (~10x) fluorescence at decreasing pH (Kapellos et al., 2016). We developed non-toxic conditions to surface label various bacterial species with pHrodo®, thus enabling a quantifiable mode for the dynamic determination of bacterial internalization. Further, the pHrodo®-labeled bacteria remain fully viable and continue to serve as functional sources for chemoattraction of *Dicytostelium* (Meena and Kimmel, 2017).

**Figure 4:**
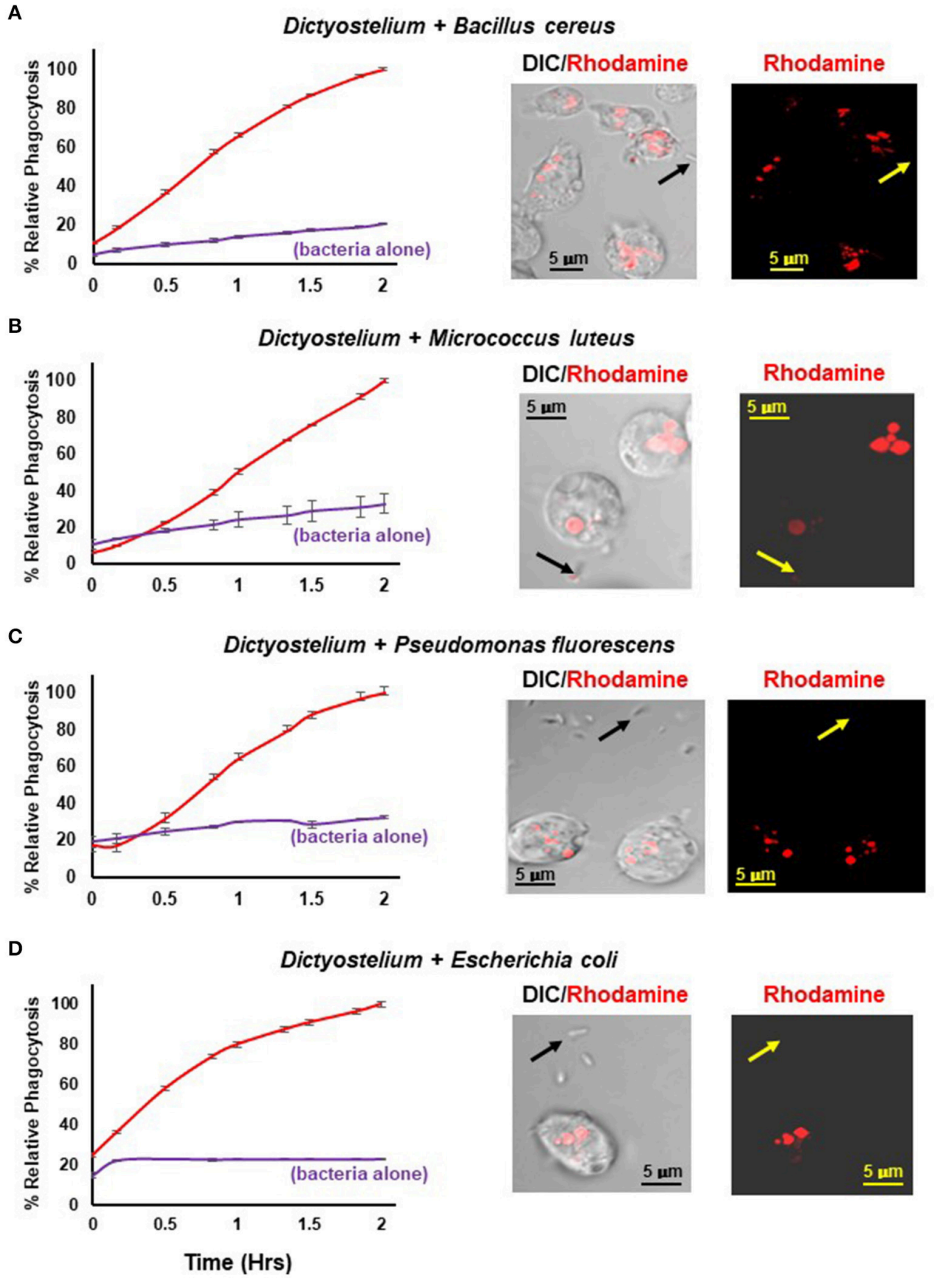
Quantified phagocytosis of live bacteria. Left panels show time-course phagocytosis quantification by WT *Dictyostelium* of pHrodo®-labeled **(A)** *Bacillus cereus*, **(B)** *Micrococcus luteus*, **(C)** *Pseudomonas fluorescens*, and **(D)** *Escherichia coli*; bacteria-alone controls are included for each. Standard deviations are shown based upon three replicates and normalized to values of 2 h. Right panels show confocal images for DIC and rhodamine fluorescence after 2 hrs of phagocytosis. Arrows indicate pHrodo®-bacteria with low level fluorescence outside of *Dictyostelium*. **(A)** *Bacillus cereus*, **(C)** *Pseudomonas fluorescens*, and **(D)** *Escherichia coli* are rod-like, whereas **(B)** *Micrococcus luteus* is coccoid; at an increased magnification, their typical rounded, clumped-cell characteristic is apparent.

Figure 4 shows a time-dependent increase in fluorescence upon phagocytosis of pHrodo®-labeled gram positive and gram negative bacteria by *Dictyostelium* and their transport into acidified intracellular vesicles; in the absence of *Dictyostelium*, fluorescence remains comparatively low throughout the time-course. The functional difference in fluorescent intensity is well visualized by confocal microscopy (Figure 4). Extracellular bacteria have very low level fluorescence, whereas, following phagocytosis, intracellular vesicles of *Dictyostelium* become progressively, highly, and specifically fluorescent (Meena and Kimmel, 2017).

### 3.4. Macropinocytosis of pHrodo®Red dextran by growth phase *Dictyostelium*

#### 3.4.1. Macropinocytosis of pHrodo®Red dextran using immunofluorescence microscopy

**3.4.1.1.** Follow all steps in **3.1.1**. to obtain growth phase *Dictyostelium*.
**3.4.1.2.** As per phagocytosis, macropinocytosis by *Dictyostelium* can be monitored by assessing fluorescence changes of pHrodo coupled to dextran, 10K, upon cellular internalization. For confocal microscopy, first add 300 μL of *Dictyostelium* at 1.0 × 10^6^ /mL in PB to a well of an 8-well Lab-Tec chambered slide and incubate at 22°C for 15 min, to allow cells to adhere. Subsequently, add 300 μL of pHrodo-Red Dextran at 30 μg/mL.
**3.4.1.3.** DIC and fluorescent [Excitation/Emission (nm) 560/585] images were acquired at varying times by confocal/multiphoton microscopy using a 100x oil objective lens and processed using Zeiss ZEN software.

#### 3.4.2. Quantification of macropinocytosis of pHrodo-Red dextran

**3.4.2.1.** ~0.9 × 10^5^ *Dictyostelium* from **3.1.1**. in 100 μL PB were added to each phagocytosis/macropinocytosis plate well and allowed to adhere to the well base for 1–5 min.
**3.4.2.2.** 100 μL of pHrodo® Red Dextran suspension in PB (0–4 μg) was then added to each well. In parallel, for negative controls, three wells had pHrodo® Red Dextran, without added *Dictyostelium*.
**3.4.2.3.** Transfer the plate to the IncuCyte chamber, at 22°C. Using a 10x objective lens, collect cell images in the visible and rhodamine detection [Excitation/Emission (nm) 560/585], a desired time (e.g., 1.5–4 h), at 15 min intervals. Export the data into Microsoft Excel for processing.

#### 3.4.3. Results

Certain cells of the immune system (e.g., macrophage and dendritic) utilize macropinocytosis in a central role for antigen uptake, processing, and presentation during pathogen defense (Lim and Gleeson, 2011; Marques et al., 2017). In *Dictyostelium*, macropinocytosis plays a role for nutrient uptake (Bloomfield et al., 2015). Macropinocytosis, however, is a distinct pathway to phagocytosis (Lim and Gleeson, 2011; Levin et al., 2016; Marques et al., 2017); the former functions for cellular uptake of fluid-phase elements, whereas the latter is focused to large particle recognition. Still, the macropinosome and phagosome systems have certain commonality with the endosomal/lysosomal pathway and vesicle acidification (Lim and Gleeson, 2011; Levin et al., 2016; Marques et al., 2017). Therefore, we utilized the pH-sensitive probe pHrodo® dextran 10K that has minimal fluorescence at neutral pH, but displays dramatic enhancement of fluorescence as the pH decreases during macropinocytosis, with endosome maturation, and fusion with lysosomes.

**Figure 5:**
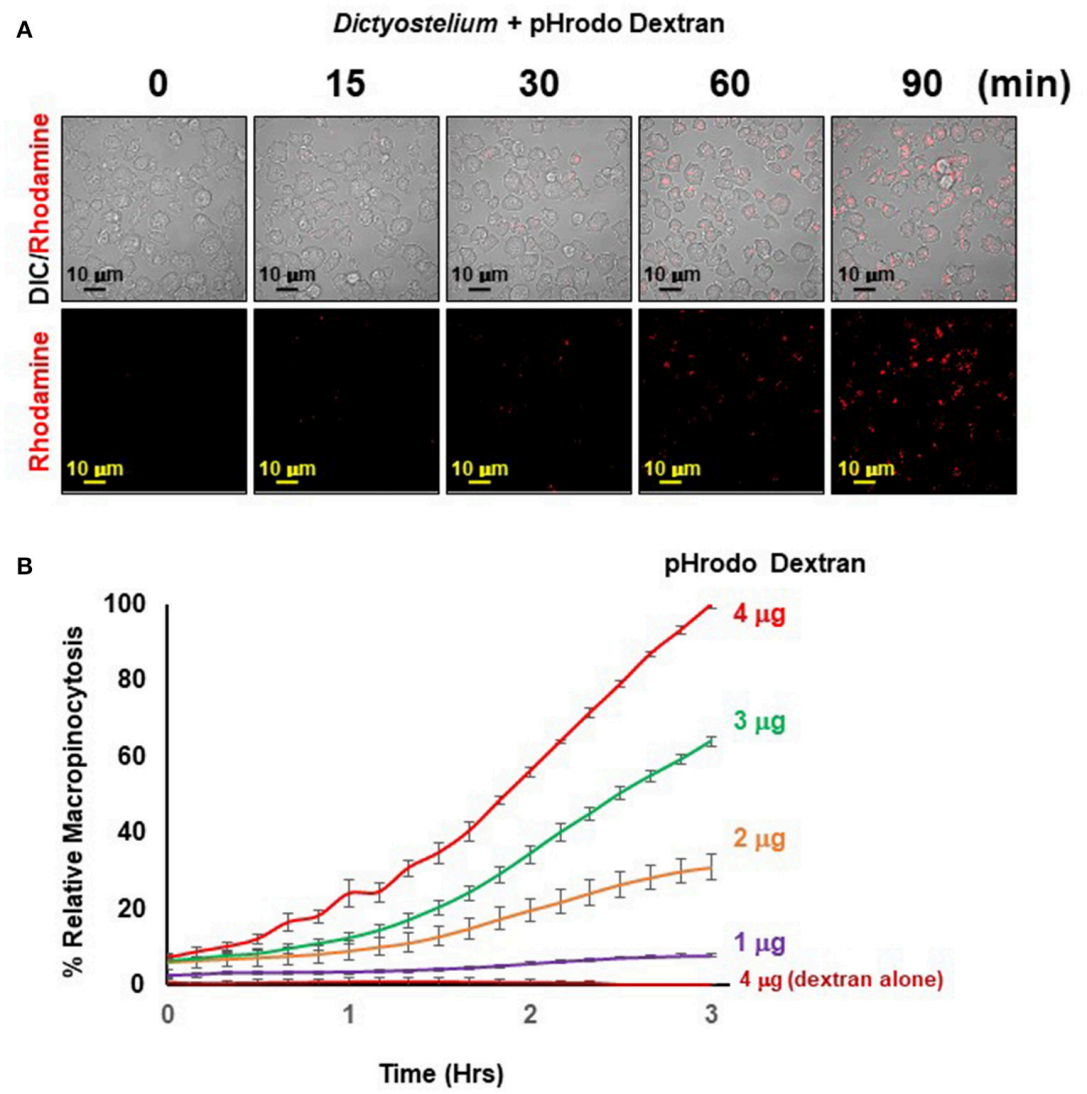
Quantified macropinocytosis by *Dictyostelium*. **(A)** DIC and rhodamine fluorescent images of pHrodo®-labeled Dextran (in red) engulfed by WT *Dictyostelium* over time. **(B)** Time-course macropinocytosis quantification by WT *Dictyostelium* of pHrodo®-labeled Dextran; a dextran-alone control is included. Standard deviations are shown based upon three replicates and normalized to values for 4 μg pHrodo®-Dextran at 3 h.

The principle of the macropinocytosis assay perfectly parallels that of phagocytosis and is, thus, readily observed by confocal fluorescence microscopy (Figure 5A) or by high-throughput measurement, where, following a lag, there is a time-dependent increase in fluorescence upon macropinocytosis of pHrodo®-labeled dextran by *Dictyostelium* and transport into acidified intracellular vesicles is highly quantifiable (Figure 5B). In the absence of *Dictyostelium*, fluorescence of pHrodo®-labeled dextran remains unchanged.

The observed kinetics do differ from that generally observed using FITC-dextran and shaking assays, where uptake is linear for ~120 min, which then plateaus. Several physical parameter distinctions underlie these kinetic differences. With FITC-dextran, intracellular fluorescence can be detected upon immediate uptake, whereas pHrodo®-labeled dextran must first traffic through acidic vesicles to become fluorescent. In suspension assays, *Dictyostelium* are exposed to continuous and non-varying concentrations of FITC-dextran, from 0 time. In the IncuCyte system, *Dictyostelium* are first adhered at 0 time, before pHrodo®-labeled dextran is added; chemical diffusion and cell movement can lead to a further time-lag for maximal dextran-cell interactions. Collectively, these factors contribute to non-linear (lag phase) curves during initial stages, and relative kinetic delays throughout the time-course. Following the lag, we see linearity for ~120 min, which begins to plateau for the lower pHrodo®-labeled dextran input ratios.

## 4. Discussion

We have applied a high-throughput platform for quantified time-course assays of chemotaxis, phagocytosis, and macropinocytosis. Although we developed these for *Dictyostelium*, they are easily adaptable to phagocytic cells of any species. The 96-well format permits pharmaceutical or small molecule evaluations for each, with sufficient scale for multiple controls and replicates. As each process is unique, their combined and distinct set of quantitative assay parameters must be considered when comparing to other approaches. Thus, quantifications rely on collective cellular response, and cannot simply separate the contribution of each individual component. While we have had success with continuous assay in excess of 6 h, we generally limit data collection to <2 h, where physiological differences between cells at their start and end points are minimized. We have confirmed utility for genetic screens in network pathway testing (Meena and Kimmel, 2017) and, thus, identified specific receptors, G proteins, etc. as essential or non-essential components in these pathways.

Although the chemotaxis data provide direct quality proof for high sensitivity at <1 nM chemoattractant concentrations, the data have significant complexity to interpretation, which differ for purified ligands or live bacteria as the chemoattractant source. Whereas the system does not provide direct quantification of migratory speed, directionality, or cell-shape change with comparison of WT and mutant cells as do single-cell, time-course chemoattractant response assays, it presents an effective complementary alterative that is highly quantifiable, in a high-throughput manner. It is, for example, highly effective to analyze response to live bacteria, receptor affinity response, essential pathway genes, novel chemoattractants, and small molecule targets, where multiple independent replicates and controls require precise temporal parallel evaluations.

After careful evaluation of diverse experiments and algorithms involving multiple replicates and controls, we conclude that precise normalizations provide the most consistent and reproducible data for interpretation, in comparison to scoring absolute numbers of migratory cells. Values for relative chemotaxis reach a plateau with time, independent of chemoattractant concentration, or bacterial numbers used [see Figures 1, 3 (Meena and Kimmel, 2017)]. Nonetheless, this is not simply because there are no migratory cells remaining in the upper well chamber at the time of the plateau. Several other factors are contributory. First, for chemotaxis, we image the number of cells associated with the bottom of the transmembrane at a given time point. However, this association is transitory, and eventually cells will drop off the membrane into the bottom well and be lost to the chemotaxis cell count. Even as new cells continuously reach the membrane, they are in balance with the cell numbers that have become dissociated, thus contributing to a chemotactic plateau appearance. Second, *Dictyostelium* continuously secrete enzymes that inactivate chemoattractants folate or cAMP; a deaminase (Pan and Wurster, 1978) oxidizes the essential amino group at position 2 of the pyrimidine ring (see Figure 1), while a phosphodiesterase converts cAMP into 5′-AMP (Franke and Kessin, 1992; Brzostowski and Kimmel, 2006). As these inactivating enzymes accumulate, they can create a buffer area around migrating cells that reduces chemoattractant gradient strength stimulation of naïve cells, and, thus, reduce their chemotactic response (Tweedy et al., 2016; Meena and Kimmel, 2017).

The chemoattractant gradients established are neither linear nor static, with variations occurring in both signal strength and gradient steepness. Initially, for ligand chemotaxis, concentrations detected by cells in the upper well chamber are relatively low, but with a very steep chemoattractant gradient. However, with time, as the ligand concentration in the upper well rises progressively, the steepness of the gradient diminishes in parallel. For bacteria as chemoattractant sources, it is even more complex. The available levels of chemoattractant is ever increasing, with their continued secretion by bacteria and accumulation in the wells of the lower chamber altering the shape of the gradient.

Apart from dissecting mechanistic pathways for chemotaxis, we are also interested to identify the chemical composition of bacterially secreted chemoattractants. We have applied several approaches to this. The mixing of saturating levels of any specific chemoattractant with cells plated in the upper well chamber will desensitize cells for chemotactic response to that same chemoattractant, but will not compete with unrelated chemoattractants in the bottom chamber wells (Meena and Kimmel, 2017). Alternatively, we have used *Dictyostelium* lacking components of known pathways to determine the presence or absence of known chemoattractants. Finally, we applied physio-chemical assays of bacterially secreted compounds for potential to identify novel molecules. Other factors need to be considered for cellular migration to bacteria that secrete more than a single type chemoattractant. Although folate and cAMP bind and activate different G protein coupled receptors, their downstream intracellular paths converge (Liao et al., 2013; Meena and Kimmel, 2017). Thus, although low sub-saturating levels (e.g., 1 nM) of folate and cAMP elicit greater chemotactic response when provided in combination than with either alone (Meena and Kimmel, 2017), no differences are seen between a single or mixed ligand preparation when either or both are at signal saturation (e.g., 1 μM). Accordingly, if a bacterial population can secrete multiple chemoattractants to saturating levels overall cell migration might remain unaltered, even if signal response were fully suppressed for one ligand.

Chemotaxis orients cell movement toward component targets for directed removal and destruction. Since phagocytosis serves as a primary process for this latter cellular function, we developed an engulfment assay of live bacteria that would precisely parallel chemotaxis and allow co-analyses to define common and unique pathways. We labeled bacteria with the rhodamine based probe pHrodo® that exhibits enhanced fluorescence with decreasing pH, and quantified phagocytic rates as a function of an increase in fluorescence, upon the internalization of pHrodo®-bacteria and trafficking into acidified intracellular vesicles. The phagocytosis assay is highly quantitative in real-time, and the 96-well format facilitates the dissection of molecular pathways, and evaluation of sensitivity to pharmaceutical agents.

Our labeling conditions do not compromise bacterial viability and pHrodo®-bacteria remain strongly chemoattractant for *Dictyostelium* (Meena and Kimmel, 2017). Moreover, we have demonstrated that *Dictyostelium* lacking chemosensitivity to pHrodo®-bacteria, through the loss of chemoattractant receptor function, retain full phagocytic capacity, thus, separating essential dependency of these cellular processes (Meena and Kimmel, 2017).

We have further demonstrated cell-viable labeling and quantitative phagocytosis of multiple gram negative and gram positive bacterial species and suggest that these procedures are adaptable to virtually any bacteria, providing an excellent extension to existing tools for dynamic studies of host-pathogen interactions during chemotaxis and phagocytosis.

Macropinocytosis is another highly regulated pathway for endocytosis, which, like phagocytosis, is essential in pathogen defense (Lim and Gleeson, 2011; Marques et al., 2017). Although both phagocytosis and macropinocytosis are actin-dependent pathways, they separately involve many distinct molecular elements (Lim and Gleeson, 2011; Bloomfield et al., 2015; Levin et al., 2016; Lim et al., 2017; Marques et al., 2017). Macropinocytosis is well-coordinated to signaling response for cytoskeletal remodeling for fluid phase uptake and, although many components and regulators are known, their precise sequence in event activations is still poorly defined. Since most macropinocytosis assays have limited real-time and/or high-throughput outputs, we consequently used pHrodo®-dextran as a fluorescent read-out, as per phagocytosis. Our results have demonstrated defined conditions for highly quantitative measurements of macropinocytosis in *Dictyostelium* in a physiological, but high-throughput, setting.

We anticipate that the multiple assays described here will have significant utility in application for functional host-pathogen studies.

## Author contributions

All authors listed have made a substantial, direct and intellectual contribution to the work, and approved it for publication.

### Conflict of interest statement

The authors declare that the research was conducted in the absence of any commercial or financial relationships that could be construed as a potential conflict of interest.
